# Meeting at St. Thomas's Hospital

**Published:** 1918-12-07

**Authors:** 


### THE ASSOCIATION OF HOSPITAL OFFICERS.

### Meeting at St. Thomas's Hospital.

Members of this Association, to the number of about 80, met at St. Thomas's Hospital at three o'clock on Saturday, November 30, for the purpose of inspecting the institution, under the year's President, Mr. G. Q. Roberts, the Secretary of the hospital. A feature of the annual gathering is a religious service, and his Grace the Archbishop of Canterbury, who takes a particular interest in St. Thomas's, kindly consented to deliver the address. The chapel service, conducted by the chaplain, the Rev. Mr. Thomas, was of a homely description, and the worshippers included the Hon. Sir Arthur Stanley, the Treasurer of the hospital; Sir Arthur and Lady Newsholme (Medical Officer of the Local Government Board), and the matron of the hospital (Miss Lloyd Still).

